# Formative research for the design of a scalable mobile health program water, sanitation, and hygiene: CHoBI7 mobile health program

**DOI:** 10.1186/s12889-019-7144-z

**Published:** 2019-07-31

**Authors:** Christine Marie George, Fatema Zohura, Alana Teman, Elizabeth Thomas, Tasdik Hasan, Sohel Rana, Tahmina Parvin, David A. Sack, Sazzadul Islam Bhuyian, Alain Labrique, Jahed Masud, Peter Winch, Elli Leontsini, Kelsey Zeller, Farzana Begum, Abul Hasem Khan, Sanya Tahmina, Farazana Munum, Shirajum Monira, Munirul Alam

**Affiliations:** 1 Associate Professor, Department of International Health, Program in Global Disease Epidemiology and Control, Johns Hopkins Bloomberg School of Public Health, 615 N. Wolfe Street, Room E5535, Baltimore, MD 21205-2103 USA; 2 International Centre for Diarrhoeal Disease Research, Bangladesh (icddr,b), Dhaka, Bangladesh; 3 Ministry of Health and Family Welfare, Dhaka, Bangladesh

**Keywords:** Mobile health, Water, sanitation, and hygiene, Diarrhea, Cholera, Bangladesh, Qualitative research methods, Formative research

## Abstract

**Background:**

The Cholera-Hospital-Based-Intervention-for-7-Days (CHoBI7) is a handwashing with soap and water treatment intervention program delivered by a health promoter bedside in a health facility and through home visits to diarrhea patients and their household members during the 7 days after admission to a health facility. In a randomized controlled trial among cholera patient households in Bangladesh, the 7-day CHoBI7 program resulted in a significant reduction in cholera among household members of cholera patients and sustained improvements in drinking water quality and handwashing with soap practices 12 months post-intervention. In an effort to take this intervention to scale across Bangladesh in partnership with the Bangladesh Ministry of Health and Family Welfare, this study evaluates the feasibility and acceptability of mobile health (mHealth) programs as a low-cost, scalable approach for CHoBI7 program delivery.

**Methods:**

Formative research for the development of the CHoBI7 mHealth intervention included 40 semi-structured interviews, 4 mHealth workshops, 2 group discussions, and a pilot study of 52 households to assess the feasibility and acceptability of the developed mHealth program. Thematic analysis of the interviews and group discussions was conducted by two individuals separately based on emergent themes, and then themes were compared and discussed.

**Results:**

A theory- and evidence-based approach using qualitative research methods was implemented to design the CHoBI7 mHealth program. Semi-structured interviews with government stakeholders identified perceptions and preferences for scaling the CHoBI7 mHealth program. Group discussions and semi-structured interviews with diarrhea patients and their family members identified beneficiary perceptions of mHealth and preferences for CHoBI7 mHealth program delivery. mHealth workshops were conducted as an interactive approach to draft and refine mobile message content based on stakeholder preferences. The pilot findings indicate that the CHoBI7 mHealth program has high user acceptability and is feasible to deliver to diarrhea patients that present at health facilities for treatment in Bangladesh. Both text and voice messages were recommended for program delivery. Dr. Chobi, the sender of mHealth messages, was viewed as a credible source of information that could be shared with others.

**Conclusion:**

This study presents a theory- and evidence-based approach that can be implemented for the development of future water, sanitation, and hygiene mHealth programs in low-resource settings.

**Electronic supplementary material:**

The online version of this article (10.1186/s12889-019-7144-z) contains supplementary material, which is available to authorized users.

## Background

Diarrhea is a leading cause of death in young children globally, causing an estimated 500,000 deaths annually [1]. Lack of handwashing with soap and water treatment are important risk factors for pediatric diarrheal disease [2–4]. Globally, only 26% of the world’s population is estimated to wash their hands with soap after coming into contact with human excreta [5]. Previous work in Bangladesh found that handwashing with soap before eating and feeding a child measured by structured observation was less than 1% [6]. Water, sanitation, and hygiene (WASH) interventions promoting household chlorination of drinking water, safe water storage, and handwashing with soap have the potential to reduce diarrheal disease incidence in children less than 5 years of age an estimated 30 to 75% [7]. However, there has been limited success in encouraging households to sustain these behaviors over time [8, 9]. Community-based WASH interventions are expensive and often difficult to implement effectively in an urban context in low-resource settings [10]. Effective scalable WASH interventions are urgently needed to reduce diarrheal diseases globally.

Phone-based reminders are an emerging low-cost intervention approach that has been shown to lead to significantly improved case management and disease prevention practices [11–15]. Furthermore, mobile health (mHealth) presents a scalable approach for which messages can be sent to a large number of households at minimal cost and can serve as valuable cues to action to facilitate behavior change. Phone access and ownership have grown tremendously with mobile phone subscriptions worldwide more than doubling over the past 10 years [16]. In 2017, there were estimated to be 85 million unique mobile subscribers in Bangladesh--half the country’s population [17]. This presents an ideal opportunity for mass mobile phone messaging of public health information. However, there are no published studies, to our knowledge, evaluating delivery of WASH intervention programs using mobile health.

Most WASH programs focus solely on educational modules related to diarrhea prevention, and do not tailor their interventions to their target population [18]. This approach does not take into account the psychosocial, technological, and contextual factors in a given setting that could influence behavior. There is a growing evidence base demonstrating interventions that use health theory are likely to yield greater behavior change than those based on health education alone [19–24]. Theory-based interventions are guided by behavior change theories and models that provide a framework for which interventions can be delivered; examples include the Theory of Planned Behavior, Protection Motivation Theory, the Integrated Behavioural Model for Water, Sanitation, and Hygiene, the Health Belief Model, and the Risks, Attitudes, Norms, Abilities, and Self-regulation (RANAS) Model [25–29]. In Contzen et al. which utilized the RANAS model, significant psychosocial factors related to handwashing with soap practices were identified at baseline and then targeted in a subsequent behavior change intervention conducted in Ethiopia [30]. This theory-based intervention resulted in significantly more handwashing with soap at stool related events than health education focused messages only [21]. However, despite this growing evidence, the use of theory-based behavior change techniques for large scale WASH programs is relatively rare [31].

### CHoBI7 program

Household members of diarrhea patients are at a much higher risk of developing diarrheal diseases (> 100 times for cholera) than the general population during the 1-week period after the diarrhea patient presents at a health facility [32–34]. This is likely because of a shared contaminated water source and poor hygiene practices [33, 35]. However, despite this high risk there is no standard of care for the household members of diarrhea patients. Furthermore, the time patients and their household members spend at a health facility for the treatment of diarrhea presents the opportunity to deliver WASH interventions when perceived severity of diarrhea and perceived benefits of water treatment and handwashing with soap is likely the highest [36]. Therefore, in an effort to develop a low-cost standard of care for this population during this 1-week high-risk period, we recently developed a 7 day health facility-based handwashing with soap and water treatment program entitled CHoBI7 (Cholera-Hospital-Based-Intervention-for-7-Days). “Chobi” means picture in Bangla. This name was selected for the pictorial WASH modules delivered as part of this intervention program. The CHoBI7 program includes: (1) a pictorial module delivered using a flipbook on how diarrheal diseases spread, and recommendations on proper handwashing with soap and water treatment; and (2) a diarrhea prevention package containing: chlorine tablets for water treatment, soapy water bottles (a low-cost alternative to bar soap made using detergent and water [37]), a handwashing station, a water vessel with tap and lid, and a sticker and cue cards on handwashing with soap and water treatment. A trained health promoter delivers this pictorial module and diarrhea prevention package to diarrhea patients and their accompanying family members during a consultation session bedside in a health facility [38]. This module is then reinforced through 30 min home visits during the 1 week period of highest risk for cholera infection among household members of cholera patients.

### Previous randomized controlled trial of the CHoBI7 intervention

To evaluate the efficacy of the targeted 7-day CHoBI7 WASH intervention in reducing cholera among household members of cholera patients during their 1-week high-risk period, we conducted a RCT of CHoBI7. We compared the CHoBI7 WASH intervention to the standard recommendation given in Bangladesh to diarrhea patients at discharge on oral rehydration solution (ORS) use. Delivery of the 7-day CHoBI7 intervention resulted in a 47% reduction in overall cholera infections, and a significant reduction in symptomatic cholera during the 1-week high-risk period [38]. Consistent with these findings, the odds of handwashing with soap at food and stool related events during structured observation was 14 times higher in the CHoBI7 arm compared to the standard recommendation arm, and 94% of CHoBI7 households had free chlorine concentrations in stored drinking water greater than the CDC recommended cut-off of 0.2 mg/L [39]. There were also no stored drinking water samples in the CHoBI7 intervention arm with *V. cholerae.* Furthermore, the 7-day CHoBI7 intervention led to significant sustained improvements in household stored drinking water quality and observed handwashing with soap practices up to 12-months post intervention [40]. These findings demonstrate that the CHoBI7 intervention presents a promising standard of care for cholera patient households during their 1 week high risk period and can result in sustained WASH practices over time.

### Theory based approach for development of the CHoBI7 intervention program

Using a theory-based approach, the CHoBI7 intervention program was informed by components of Protection Motivation Theory, IBM-WASH, and the RANAS Model [25, 27, 29]. Using these theories and models, we developed behavior change techniques for the CHoBI7 intervention program targeting remembering, perceived susceptibility and severity, cholera awareness, disgust, response efficacy, convenience, and self-efficacy (based on previous studies [18, 21, 30, 41–43]), and used Likert scale statements to measure these psychosocial factors (methods are published in George et al. [44]). By measuring these psychosocial factors during our recent RCT of CHoBI7, we were able to investigate the underlying mechanism of change that led to the high handwashing with soap behavior observed among intervention participants. Response efficacy (judgments about the efficacy of a preventive response that will avert the perceived threat [29]) was found to mediate the intervention’s effect on handwashing with soap habit formation at the 1-week follow-up, whereas disgust, convenience, and cholera awareness were mediators of habit maintenance at the 6 to 12-month follow-up [44]. Our study was the first RCT of a WASH intervention that conducted a mediation analysis to investigate the underlying mechanism of change in a low-income country.

### Rationale for the development of the CHoBI7 mobile health program

Based on this previous work, the Bangladesh Ministry of Health and Family Welfare has expressed an interest in including the CHoBI7 program in the upcoming National Operational Plan for Communicable Disease Control, and has requested evidence be provided on low-cost, scalable approaches that could be implemented to deliver the CHoBI7 program across Bangladesh. Therefore, this current study building on our previous work evaluates the feasibility and acceptability of implementing a mHealth program as a low-cost, scalable approach for CHoBI7 program delivery that does not involve frequent in-person visits. This paper reports the formative research findings from this study, which have informed the design of the CHoBI7 mHealth program. The primary objective of this formative research was to develop a WASH mHealth program that could be taken to scale by the Bangladesh Ministry of Health and Family Welfare to serve as cues to action to facilitate sustained WASH behavior change.

In this study we take a theory- and evidence-based approach using qualitative research methods to design and tailor the CHoBI7 mHealth intervention program for our target population in Bangladesh. The CHoBI7 mHealth program developed through these formative research activities is currently being evaluated in a RCT. This is the first RCT, to our knowledge, evaluating the impact of a WASH mHealth program.

## Methods

### Formative research activities

**Fig. 1:**
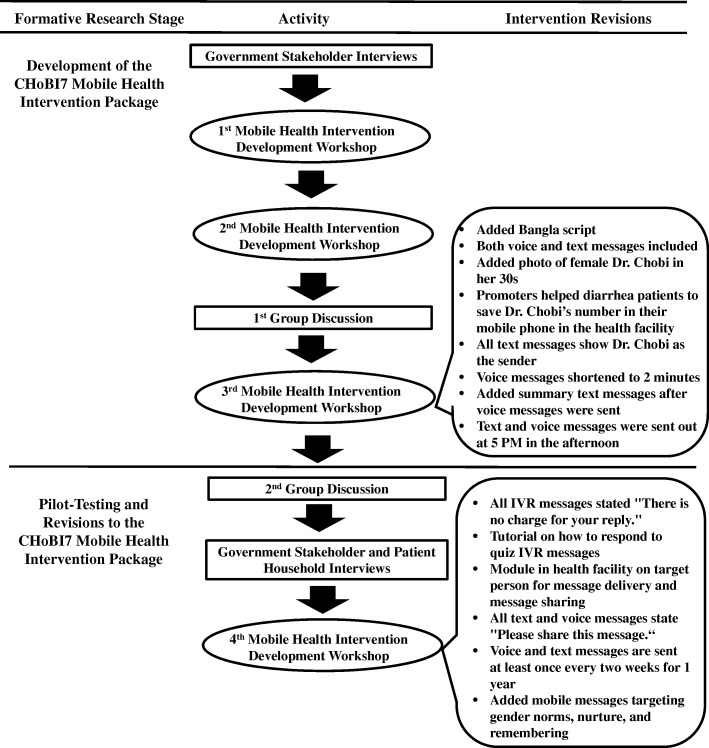
Formative Research Activities for CHoBI7 Mobile Health Program Development

The formative research activities had three components: (1) intervention development through mHealth workshops; (2) semi-structured interviews and group discussions; and (3) a pilot study of the CHoBI7 mHealth program. An overview of formative research activities is shown in Fig. 1. Research activities were a partnership between the Johns Hopkins Bloomberg School of Public Health, the Bangladesh Ministry of Health and Family Welfare, and the International Centre for Diarrhoeal Disease Research, Bangladesh (icddr,b). The formative research aims were the following: (1) identify government stakeholders perceptions and preferences for scaling the CHoBI7 mHealth program in Bangladesh; (2) identify beneficiary perceptions and preferences for delivering this program (e.g. mobile message type, message content, and message timing and frequency); and (3) determine the feasibility of implementing this program (e.g. mobile phone access and message sharing).

#### Intervention development

The CHoBI7 mHealth intervention program development targeted five key behaviors: (1) preparing soapy water using water and detergent powder; (2) handwashing with soap at food- and stool-related events; (3) treating household drinking water using chlorine tablets during the one-week high-risk period after the diarrhea patient in the household was admitted to the health facility; (4) safe drinking water storage in a water vessel with cover; and (5) heating of household drinking water until it reaches a rolling boil after the one-week high-risk period. The CHoBI7 mHealth program was developed through a theory-based approach, which was informed by IBM-WASH and Protection Motivation Theory [25, 29]. Intervention development was guided by IBM-WASH to target the habitual, individual, interpersonal/household, community, and structural/societal-level factors that are drivers of our target WASH behaviors [25]. For example, higher-level contextual and technological factors, such as national policies on WASH and delivery of mHealth messages to the public were considered through the engagement of government stakeholders in intervention development; and habitual level factors such as frequency of exposure to mobile messages were explored by engaging pilot participants in interviews and discussion groups. This multi-level approach allowed us to develop behavior change techniques to regulate factors identified during formative research that facilitated or impeded the targeted behaviors.

mHealth messages were developed to target the following psychosocial factors: response efficacy of the behavioral recommendations, remembering of handwashing with soap, water treatment, and safe water storage, convenience (e.g. promoting the use of enabling hardware), awareness of diarrhea transmission and prevention, nurture towards children in the household, self-efficacy of behavioral recommendations, feelings of disgust towards feces, perceived severity and susceptibility, and gender norms and roles (e.g. acceptability of the female household members receiving mHealth messages) [25, 29]. Table 1 includes examples of the mHealth Behavior Change Techniques included in the CHoBI7 mHealth program to target these psychosocial factors and IBM-WASH dimensions. These psychosocial factors were identified through: (1) the formative research findings presented in this publication (gender norms/roles, nurture, and remembering); (2) psychosocial factors associated with habit formation and maintenance in our previous RCT of the CHoBI7 intervention (response efficacy, disgust, convenience, and diarrhea disease awareness) [44]; and (3) factors found in previous studies to be associated with WASH behavior change (self-efficacy and descriptive norms [25] and perceived severity and susceptibility [29, 30]).

**Table 1:** Example of Theory-based Approach for Development of CHoBI7 mHealth Messages: Psychosocial Factors and IBM-WASH Dimensions

Psychosocial Factor	Factor Definition	Behavior Change Technique	IBM-WASH Dimension	Example Messages	Hypothesized Change with Intervention
Self-Efficacy	The belief in one’s capabilities to organize and execute the courses of action required to manage prospective situations. (Bandura et al. 1997)	mHealth messages providing information on actionable tasks	Structural (Gender Roles)/ Individual	Voice Message *Dr. Chobi Apa: How are you brother? How is Aklima and your children?*	*Higher acceptability of WASH behavioral recommendations among men*
				*Husband: Yes, good! My children got frequent diarrhea and sometimes we needed to hospitalize them before. Now, they remain healthy.*	*Higher self-efficacy among male household members*
				*Dr. Chobi Apa: Why do you think they remain healthy now?*	
				*Husband: Because my family and I now boil and safely store our drinking water and wash our hands with soapy water after defecation, after cleaning a child’s feces and anus, before preparing food, and before eating or feeding children. See, you phoned and texted me which I have shared with my family and followed all the instructions.*	
		mHealth messages encouraging the use of enabling technology	Habitual	Text Message *To keep your water safe to drink, always put your boiled water in the blue bucket with the lid on. Never put your hands into this water, always use the tap. Keep your family happy and healthy*!	*Higher self-efficacy and convenience of WASH behavioral recommendations*
Descriptive Norms	Perceptions about which behaviors are typically performed by others (Cialdini et al. 2006)	mHealth messages describing the proportion of others in the community performing the same behavior	Community	Voice Message *I am Dr. Chobi Apa from Mohakhali Cholera Hospital. How are you? We have found that 80% of individuals in your neighbourhood reported washing their hands with soap before feeding their child this week. Like others in your neighborhood, always wash your hands with soap before preparing food and feeding your child. Make sure there is always soapy water present within 10 steps of your cooking area. Keep your family healthy and happy!*	*Higher descriptive norms of WASH behavioral recommendations*
		mHealth messages from Aklima, a woman who brought her child to a health facility for diarrhea treatment, and who learned proper handwashing and water treatment behaviors from Dr. Chobi.	Interpersonal	Voice Message *This is Dr. Chobi Apa. Aklima is here with me again today and wants to share her story with you. Aklima: My son was very sick; he almost died. We took him to the hospital and the doctors gave him treatment. Before I left the hospital, they gave me a nice red handwashing bucket and blue water bucket. Dr. Chobi Apa told me about washing my hands with soap at four key times: before I prepare food, before I eat, and after I use the toilet or clean my child’s feces or anus. I have followed all these instructions all the time, and my family is now healthy and happy!* *Dr. Chobi: Thank you Aklima for sharing your story! Did you hear how well Aklima is doing now? Are you also practicing these safe practices and staying healthy?*	*Higher descriptive norms of WASH behavioral recommendations*
Emotion of Nurture	The desire to care for someone, and see them develop or grow.	mHealth messages encouraging nurture of young children as a motivator to practice the key behaviors promoted	Interpersonal	Voice Message *Diarrhea especially hurts small children.* *To protect your child from these germs, make sure your whole family washes their hands at 4 key times: after defecation, after cleaning a child’s feces and anus, before preparing food, and before eating or feeding children. Use soapy water each time!*	*Higher nurture for young children*
Emotion of Disgust	Revulsion that is occasioned by the sight of excreta, rotten food, slime, and bugs. (Curtis et al. 2001)	mHealth messages encouraging the emotion of disgust as a motivator to practice the key behaviors promoted	Individual	Text Message *If you don’t wash your hands after using the toilet, you’re eating feces when you’re eating your food. There are lots of germs in feces, and you may have diarrhea again.*	*Higher disgust*
Remembering	To perform a behavior, it has to be remembered at the right time/situation. (Tobias et al. 2009)	mHealth messages reminding households to perform the key behaviors promoted	Habitual	Text Message *Have you made a habit of drinking safe water and washing your hands with soap to create a shield for good health? If not, you can start now!*	*Higher daily remembering of behavioral recommendations*

The CHoBI7 mHealth messages were developed during 4 mobile health workshops. The initial workshop in June 2016 was led by a mHealth expert that trained the study team on techniques for delivering voice, text, and interactive voice response (IVR) messages. The second mHealth workshop was later in June 2016, the third was in July 2016, and the fourth in September 2016. There were 6–10 participants in each mHealth workshop, and workshops were 2 to 4 h in length. Workshop participants were CHoBI7 research team members including the study intervention and project coordinator, health promoters, and study investigators. During the workshops, contextual, psychosocial, and technological factors that emerged during the qualitative research were discussed using the IBM-WASH framework (Table 2). Mobile messages targeting these factors were than written on a flip chart by team members and discussed and refined as a group.

**Table 2:** The IBM-WASH Framework Applied to the Development of the CHoBI7 Mobile Health Intervention Based on Qualitative Findings

BM-WASH Dimension	Contextual Factors	Psychosocial Factors	Technological Factors
Structural/ Societal	• Existing government mobile health programs send out health-related messages on government health days, these include voice and text mobile messages • Potential inclusion of CHoBI7 intervention in the National Operational Plan in Bangladesh • Potential integration of CHoBI7 in existing mobile health programs in Bangladesh	• Government commitment to mobile health as a method to deliver public health information	• Existing government mobile platform has the potential to be used for CHoBI7 intervention delivery at reduced cost • Health Education Bureau in the Ministry of Health and Family Welfare currently develops mobile health messages, and can be potentially engaged for CHoBI7 intervention development
Community	• High household mobile phone access and ownership in Bangladesh	• Sharing of mHealth messages with neighbors	• High mobile network coverage in Dhaka, Bangladesh
			• Most feature phones available in Bangladesh allow for viewing of Bangla script
Interpersonal	• Females in the households are often the ones responsible for caring for young children • Male household members may not want female household members to receive text and voice messages from an unknown sender	• Female caregivers requesting access to CHoBI7 mHealth messages to allow them to better care for their children	• Text messages allow for sharing with others at a later time • Male household members do not always share mobile messages or their mobile phones with other household members • Timing for mobile message delivery when all household members are present • Adult male household members typically have primary mobile phone ownership in household • Lower female access to mobile phones
Individual	• Literacy rate of household members • Limited mobile message sharing by those working outside of the home	• Self-efficacy to open text messages, and respond to interactive voice response messages	• Concerns about being charged a fee for viewing or listening to mobile messages
Habitual	• Frequency of exposure to mobile messages	• Outcome expectancy that following recommendations contained in mobile messages will reduce disease	• Voice and text messages as reminders to perform the promoted water, sanitation, and hygiene behaviors

The VIAMO platform was used to deliver text, voice, and IVR messages to pilot households (https://viamo.io/). IVR messages were sent as “quiz” questions for recipients to assess their knowledge of the WASH key behaviors recommended by the CHoBI7 intervention. There was no call- or text-back option available, or hotline.

#### CHoBI7 mHealth pilot

The objective of the pilot study was to identify the feasibility of implementing the CHoBI7 mHealth program, and potential challenges for successful program implementation. Fifty-two households of diarrhea patients (7 Control Arm, 23 mHealth with no home visits arm, and 22 Intervention Arm 2) were enrolled in the pilot study of the CHoBI7 mHealth program. Diarrhea patients presenting at icddr,b Dhaka Hospital or Mugda Hospital in Dhaka, Bangladesh with three or more loose stools over a 24 h period were recruited for the pilot study. Households of diarrhea patients were eligible for the study if they had a child under 5 years of age and at least one household member owned an active mobile phone. Household members were defined as those individuals sharing the same cooking pot with the diarrhea patient for the past three days. Households were followed up to 3 months. The Control Arm (Standard Message Arm) received the standard message in Bangladesh given to diarrhea patients on the use of ORS (Additional file 1: Table S1). The mHealth with no home visits arm households received: (1) the standard message; (2) one visit by a promoter to deliver the CHoBI7 pictorial module during the patient’s visit for diarrhea treatment at the health facility; and (3) CHoBI7 mHealth voice and text messages at least once every two weeks. The mHealth with home visits arm households received the same activities as the mHealth with no home visits arm plus two – 30 min home visits by a health promoter during the first week of intervention delivery. A diarrhea prevention package was given to diarrhea patients in the intervention arms by promoters during treatment at the health facility. This package contained chlorine tablets for water treatment, a soapy water bottle, a handwashing station, a water vessel with lid, and a sticker and cue cards on handwashing with soap and water treatment. Participants were assigned to study arms based on the day of the week they were admitted to each health facility. The pilot study was conducted in an iterative manner where changes to the CHoBI7 mHealth message content and delivery were made based on participant feedback from semi-structured interviews and group discussions between May to November 2016.

#### Semi-structured interview and group discussions

Forty semi-structured interviews were conducted from May to November 2016. Respondents were selected through convenience-based sampling [45]. There were 12 government stakeholder interviews to identify government stakeholder perceptions and preferences for scaling the CHoBI7 mHealth program in Bangladesh; and 28 intervention arm pilot participant interviews to identify beneficiary perceptions and preferences for delivering this program and to determine the feasibility of implementing this program. Government stakeholders were selected if they were involved in mHealth or WASH activities at the Bangladesh Ministry of Health and Family Welfare. All government stakeholders were responsible for the implementation of government programs related to health in Bangladesh. Participants from diarrhea patient households were selected based on willingness to participate and availability of time. One pilot household participant was interviewed twice. All pilot participant interviews were in Bangla. Eleven government stakeholder interviews were in English and one was in Bangla. One government stakeholder responsible for health policy on diarrhea prevention in Bangladesh was interviewed three times from May to September 2016. During government stakeholder interviews the flipbook and diarrhea prevention package were shown, and feedback was requested. Thirty-nine of the interviews were audio-recorded, one respondent asked to not be recorded. Twenty three of the semi-structured interviews were transcribed verbatim, and summary field notes were written for 39 interviews. One interview could not be transcribed because of the poor quality of the recording. The transcripts and recordings for the pilot study interviews were used to develop a summary framework focused on the key research questions for the analysis. The summary framework was then completed for all pilot participant interviews.

Two group discussions were conducted. The first group discussion had 7 participants and the second group discussion had 12 participants. These individuals were selected using convenience sampling. Both group discussions included diarrhea patients admitted to icddr,b Dhaka Hospital and Mugda Hospital in the past 3 months and household members of these diarrhea patients. Participants in the first group discussion, did not have previous exposure to our CHoBI7 mHealth messages. The objective of the first group discussion was to obtain stakeholder feedback on the mobile messages that had been developed during the initial two mhealth workshops. These messages are shown in Additional file 1: Table S2. This feedback was used to refine mobile messages before delivery in the pilot study. All participants in the second group discussion resided in households that had participated in the pilot of the CHoBI7 mHealth program and had received these voice and text messages for up to 6 weeks prior to the group discussion. The objective of the second group discussion was to assess the usability and acceptability of the developed mHealth messages delivered in the pilot. A notetaker summarized the feedback from both group discussions.

The semi structured interviews and group discussions were conducted with a guide covering the following topics: current sources of health related information, experiences with the current and previous mobile health programs, and recommendations for delivery of CHoBI7 WASH mHealth program. Thematic analysis for the interviews and group discussions was conducted by two individuals separately based on emergent themes. Codes based on emergent themes and the IBM WASH framework were then compared and discussed. The themes that emerged where put in the following categories: current mobile health activities in Bangladesh, use of mobile phone messages for CHoBI7 program delivery, mobile message type preference for CHoBI7 mHealth program, sender of CHoBI7 mHealth program messages, message delivery and content, access to CHoBI7 mHealth messages, and timing and frequency for CHoBI7 mHealth message delivery.

## Results

### Bangladesh Government’s use of mobile health

#### Government stakeholders

All government stakeholders interviewed described previous mHealth activities implemented by the Bangladesh government for health awareness and healthcare capacity building. Most of these mHealth messages were developed in partnership with the Health Education Bureau in the Ministry of Health and Family Welfare. Respondents described a range of mHealth activities including mobile messages sent to the public during epidemic disease, and messages sent using the voice of the Prime Minster for National Immunization Day, Community Clinic Day, and World Health Day. Health call centers were mentioned where a “16263” phone number is used for the public to talk directly to doctors, and to request ambulance services for a fee. A telemedicine program was described for physicians in Dhaka to connect with patients across the country. In addition, government stakeholder stated that all government health facilities receive a mobile phone for patients to text their complaints and suggestions 24 h a day.

### Acceptability of mobile phone messages for CHoBI7 program delivery

#### Government stakeholders

All government stakeholders were receptive to the use of mobile messages for CHoBI7 program delivery. The high phone coverage in Bangladesh was said to be an advantage, allowing health awareness messages to reach many individuals across the country:

*In my country right now 90% of our people are connected with mobile phones. Using mobile phones is a very smart behavior for sharing information and knowledge. My people starting from the very poor to the high [wealthy] people you just give them the message, it is not a big challenge. They will take it*.

Text messages were mentioned to be low in cost, and could potentially be funded by the government:

*For generating text messages, funding will be required. Since the funding is not big, if external funding is available, then better. But it is possible to do [this] by the government funding.*

Government stakeholders recommended CHoBI7 mobile messages be sent through the Management Information System Department in the Ministry of Health and Family Welfare and through the Bangladesh Telecommunication Regulatory Commission. Several government stakeholders also mentioned that sending CHoBI7 mobile messages through the government system would reduce the cost of message delivery.

Government stakeholders recommended the CHoBI7 program be included in the upcoming National Operational Plan for the Communicable Diseases Control (CDC) Department at the Ministry of Health and Family Welfare. Integration of the CHoBI7 mHealth program into existing mHealth programs such as the Health Call Centers was recommended. Mobile applications were also recommended to deliver CHoBI7 messages since this would align with components already planned for the upcoming National Operational Plan. Several government stakeholders recommended that CHoBI7 mobile messages be developed in partnership with the Health Education Bureau:

*Request the Health Education Bureau, they will form the message, taking the participation [of] you and other experts … who [will] prepare this message. And [they] will disseminate this message through [out] the whole country and [the] funding required for this message will be, given from this [National] Operational Plan.*

#### Patient households

CHoBI7 mHealth messages were well-received by group discussion and pilot interview participants. CHoBI7 voice and text messages served as important reminders for patients and their household members to perform the recommended behaviors after returning from the health facility. Participants liked that the messages were coming from the hospital, and mentioned this brought credibility to the messages being sent:

*Both IVR [voice] and [text] messages are good. Many people can learn from this and by saying this information is from the hospital, we can let other people know about this information as well.*

*It was really good to get a call after coming from hospital.*

A few participants said CHoBI7 messages would be better understood and well-received through in-person visits or a combination of in-person visits and phone calls, with one participant mentioning that not all problems can be solved over the phone. A few participants recommended pictures or videos be added to mobile phone messages.

### Voice of the CHoBI7 program mHealth messages: Dr. Chobi and Aklima

#### Government stakeholders

Government stakeholders recommended a wide range of individuals to be the sender for CHoBI7 mobile messages including the Prime Minster, the Health Minister, a parliament member, a health care provider, a high level person from icddr,b, a socialite, or celebrities such as a cricket player or singer. A female voice was said to be attractive for message delivery. One respondent mentioned that the visibility of the person sending the messages was very important, and that the government is planning for people to see the person who is sending voice and text messages in future programs.

### Character development

Two characters were developed to deliver the mHealth messages: Dr. Chobi and Aklima. Dr. Chobi is a doctor at icddr,b hospital who called and texted participants to share information and reminders on handwashing with soap and water treatment behaviors. She was sometimes also called “Dr. Chobi Apa,” meaning “Sister Dr. Chobi.” Dr. Chobi introduced Aklima, a woman who brought her child to a health facility for diarrhea treatment, and who learned proper handwashing with soap and water treatment behaviors from Dr. Chobi. Aklima shared how she used Dr. Chobi’s advice to successfully keep her family safe from diarrheal disease. While Dr. Chobi was introduced as a guide, Aklima served as a peer role model for the participants designed to target descriptive norms—they could learn anything she could learn and also keep their families safe and healthy.

#### Patient households

There was a positive response for Dr. Chobi:

*I could feel her [Dr. Chobi’s] words from my heart.*

Participants in the first group discussion helped determine Dr. Chobi’s age and sex. In the first group discussion, a recording was provided of a male and female voice for Dr. Chobi. Participants liked both voices. Participants in the first group discussion were also shown a photo of a female Dr. Chobi in her 20s and a second image in her 30s. The majority of participants preferred the image of the woman in her 30s, stating she had more gravitas. Based on the feedback from the first group discussion, the Dr. Chobi in her 30s was selected as the character for the CHoBI7 program, and images of her were showed to participants in the pilot.

Participants said Dr. Chobi’s name was easy to remember, and thought that all of the CHoBI7 program messages should come from her. Participants also recommended that her name should show up on the caller ID when phone messages were sent. Voice messages were viewed as a more effective way to popularize Dr. Chobi because individuals could interact directly with her and hear her voice. Participants in the pilot requested weekly sessions with Dr. Chobi to discuss their health problems.

Aklima was also well-received, with one participant stating “I am Aklima—it’s me, not Aklima”, and another stating “It’s my life story.” Participants recommended Aklima share her knowledge with others:

*Aklima was a patient like me and it is good that she is taking help from Chobi apa [sister] like us … She should promote all this knowledge to others.*

### Text and voice messages for delivery of CHoBI7 program

#### Government stakeholders

Voice and text messages were recommended by all government stakeholders for CHoBI7 program delivery, except for one who recommended mobile messages however did not specify a type. Voice messages were recommended because they could be more easily understood than text messages by individuals that could not read, with non-literate individuals described as the ones needing the CHoBI7 program messages the most. Respondents recommended voice messages come as calls without requiring files to be opened. Text messages had been used in previous government programs, and were said to have a positive impact and be important. Phonetic Bangla was recommended for text messages because many individuals were stated to not have smart phones.

#### Patient households

The response was positive for both voice and text messages, with some participants recommending both voice *and* text messages be sent:

*Both voice and text [messages] are good. From calls we can learn and if sometimes we forget, we can see the [text] message or tell others to read them for us. That is why both together are good.*

Voice messages were viewed as better than text messages for understanding message content. While the ability to save text messages was mentioned as an advantage over voice messages that could not be saved. Text messages were also described as better for sharing message content with others:

*I can show this [text] message to 10 more people.*

Some participants said text messages were faster to read and access than voice messages. However, text messages were problematic for those that were not literate. Some participants said no one in their home could read messages aloud to them. While other participants mentioned that household members could read them CHoBI7 program text messages.

In the first group discussion, phonetic Bangla (Bangla written in English letters) was used to send CHoBI7 mHealth messages to participants based on the recommendations from government stakeholder interviews. Some Bangla words spelled using English characters such as the word for diarrhea, “patla paikhana”, were challenging for participants to understand. Participants recommended all text messages be in Bangla script so messages were more easily understood.

Participants recommended having text messages summarizing the content of voice messages (summary text messages), so if a voice call was dropped individuals could still receive CHoBI7 messages:

*If the phone [voice message] comes first and the message [text message] comes later this would be good. Then if you cannot remember the content of the call, you can read the message [text message].*

The CHoBI7 IVR quiz questions had a positive response:

*I feel so happy when Chobi apa [sister] says-yes! You are right [IVR quiz message response].*

Participants enjoyed taking these quizzes, and mentioned discussing the content of these IVR quiz messages with neighbors. Some participants described previous experiences where money was “cut” from their phones when they replied to text messages from health information lines and herbal companies. Participants mentioned being comfortable submitting quiz IVR responses because Dr. Chobi stated it was free to reply during these messages.

A few participants mentioned a preference for direct phone calls over recorded voice messages. One participant expressed concern that some people may not receive CHoBI7 voice calls because they will think they are advertisements from different companies. Some participants had full inboxes and could not receive CHoBI7 text messages, or needed to delete CHoBI7 text messages to free space in their inboxes. Additional challenges included lost phones, broken phones that didn’t allow participants to view messages, not knowing how to open text messages, and not checking text messages regularly. Some of the participants in the first group discussion mentioned that voice messages were too long and should be shorten.

### CHoBI7 mHealth message delivery and content

#### Government stakeholders

For the CHoBI7 mHealth program, government stakeholders recommended giving only a few key messages, and recommended that these messages be very specific and based on scientific evidence. This was considered important given the limited time people would likely give for the CHoBI7 program once they returned from the hospital:

*During their hospital stay they will give 80% of their time to health related issues but in their house they may give 1% of their time …[give] a few messages … what you want to emphasize.*

Government stakeholders recommended that CHoBI7 mHealth messages be integrated with other health care messages, such as those on antenatal care. Government stakeholders also recommended tailoring CHoBI7 mHealth messages to high-risk populations to ensure the messages were relevant to those receiving them. Delivering mobile messages during diarrhea outbreaks was given as an example:

*Sending messages out to everyone would be a wastage of money … [this] should be for times like during diarrhea outbreaks and should be areas-based [and] people-based not general.*

However, it was mentioned that individuals should be practicing handwashing with soap always not only during outbreaks.

#### Patient households

Message content was found to be clear and participants said messages from Dr. Chobi were stated to be culturally appropriate. Mobile messages from the CHoBI7 program were considered to be important for spreading awareness and improving health:

*Everybody will see these messages and learn from them. They would then be free from cholera and other diseases.*

During the first group discussion participants reacted strongly to Mobile Message 4 in Additional file 1: Table S2 on “eating poop.” When asked if this message content should be changed participants said these words were true and should remain:

*These are not bad words, and people will be more careful after reading the text message.* (Mobile Message 4 in Additional file 1: Table S2).

Participants understood and liked the messages promoting handwashing with soapy water, mentioning these message were important for reducing diseases:

*We always have to be clean to stay away from diseases. Like Dr. Chobi says, we should always wash our hands. We should wash our hands right after cleaning up children after toileting [defecating]. If we do this we would be free from all diseases.*

### Access to CHoBI7 mHealth messages

#### Patient households

Participants shared messages from Dr. Chobi with spouses, family members, and neighbors. Participants recommended that those receiving CHoBI7 mobile messages should share them with others including neighbors and family members to spread these messages:

*Chobi apa [sister] taught us. We are Aklima, we will tell other people … They will gain a benefit from this.*

Participants also recommended that CHoBI7 mobile messages be sent to both husbands and wives. Female participants emphasized the importance of messages from Dr. Chobi coming to their phone since they were often the ones responsible for taking care of children and would like to maintain the promoted hygiene practices:

*The person who is in charge of maintaining the home--you should send [mobile messages] to her.*

One participant said that although she has a mobile phone, these messages should be sent to her husband’s phone, because sometimes husbands may be suspicious of wives if an unknown person calls or sends them a text message. Another participant expressed concern about the CHoBI7 program potentially sending messages to his wife’s phone.

Several female pilot participants did not have their own mobile phone. Some of these participants said that their husbands or other household members shared CHoBI7 messages with them. Others reported looking at their husband’s phone to see these messages. This was often at lunch or in the evening when male household members were home. However, several female pilot participants without mobile phones said that male household members were often not sharing the content of CHoBI7 messages with them:

*If you send this information by mobile phone, it would be good, but not everyone will come to know this [information]. Like, I don’t have a mobile phone. Then how would I know that you sent a message? Suppose my husband has a mobile phone and receives and reads those [text] messages but doesn’t tell me anything about them? Don’t we have to know about these [messages]? Of course, we have to know about these messages.*

Some female participants mentioned not being able to listen to CHoBI7 voice messages because their husband had the only mobile phone in the household and was away at work when the messages were sent.

### Timing and frequency for CHoBI7 mHealth message delivery

#### Government stakeholders

One government stakeholder recommended CHoBI7 mHealth messages be sent every two weeks for a duration of three months. No recommendations were given on the timing for message delivery.

#### Patient households

Evening time was preferred by the most participants for receiving mobile messages from the CHoBI7 program. The most popular time window was 4:00–8:00 PM. This was the time when participants and their family members, including husbands, were often at home and eating and bathing:

*At night the phone remains with us. We do not go anywhere then, do not remain busy, and the phone remains nearby. If a call comes we can receive it immediately. And if a [text] message comes we can see it. So if [the message] is sent at night it would be good*.

Evening was also mentioned as an important time to stay clean. Participants described sharing text messages with each other in the evening:

*We received [text] messages at 5 pm ... We all read those together in the night.*

Most pilot participants preferred to receive CHoBI7 phone messages at least once per week, and all pilot participants preferred to receive at least one mobile phone message monthly. Frequent phone messages were considered important to make sure promoted behaviors were maintained and not forgotten, and to make individuals more aware:

*If you don’t send us text messages frequently, we will forget everything within 6 months. So, we will be able to keep them in our mind if you send them frequently.*

However it was also emphasized that messages should not be sent too frequently, and should not cause interruptions at work. One participant said that more than twice monthly could be irritating. Participants’ recommended CHoBI7 mHealth messages be sent for 6 months to 1 year.

### Intervention development

The IBM-WASH framework was applied to the development of the CHoBI7 mHealth intervention based on qualitative findings. This framework is shown in Table 2. Identifying the contextual, psychosocial, and technological factors at each IBM-WASH level has allowed us to design a mHealth intervention that targets the multi-level, multidimensional factors that are facilitating or impeding our recommended WASH behaviors. An overview of revisions made to the CHoBI7 mHealth program based on formative research findings is shown in Fig. 1. Additional file 1: Table S3 provides a detailed description of each intervention component added.

#### CHoBI7 mobile message type

Both voice and text messages are included in the CHoBI7 mHealth program based on the recommendations from interview and group discussion participants. This includes summary text messages sent out after each voice message to serve as reminders to perform the key behaviors (targeting remembering). Mobile messages are also sent out using Bangla script based on the recommendations from the first group discussion. It was found that Bangla script can be viewed on feature phones without smart phone capabilities.

#### CHoBI7 mobile message sender

Based on the recommendations of government stakeholders and participants in the first group discussion, a female health care provider, Dr. Chobi, was selected to be the sender of voice and text messages for the CHoBI7 mHealth program. Given the iterative nature of message development, using the voice of a government official was explored, however, was found not to be feasible because of the repeated recordings of voice messages that was needed. A celebrity was also found to not be a feasible option for the same reason and because of the high cost for their time.

Promoters at health facilities ask diarrhea patients and their family members to save the number for Dr. Chobi in their phones upon enrollment in the CHoBI7 mHealth program. In addition, all text messages show Dr. Chobi as the sender. This was included to ensure individuals receiving calls and text messages from the program know the sender. A tutorial is also given by health promoters in the health facility on how to open text messages and respond to an IVR quiz message to respond to concerns about difficulties opening text messages and responding to quizzes.

#### CHoBI7 mobile message delivery

In an effort to increase access to mHealth messages among female caregivers, we added a module delivered by a promoter in the health facility during the patient’s stay in the hospital for illness on mHealth message delivery and access. During this module the promoter starts by asking diarrhea patients and their family members which phones in the household should receive CHoBI7 mHealth messages. The health promoter then recommends mobile messages be sent to the phone of the primary caregiver of the child under 5 years in the home, when possible, explaining the importance of caregivers receiving these messages for the health of their children (targeting nurture). Promoters also explain that a female health provider will be contacting them by phone, never a male. This module was included to try to alleviate concerns from husbands about voice and text messages being sent to their wife’s phone, and based on recommendations to send phone messages to the female household members.

All CHoBI7 text and voice messages state “Please share this message” to promote message sharing among those in the household without personal mobile phones or not receiving program messages. This was added to address challenges with message sharing identified in the pilot. Voice and text messages are sent at 5 PM, this was recommended as a time when most individuals were home. CHoBI7 voice and text messages are sent to households at least once every 2 weeks for 1 year, based on the recommendations from interviews. Voice messages were shortened to approximately 2 minutes based on feedback from the first group discussion. This message length was reported to be fine in the second group discussion. All IVR quiz messages state “There is no charge for your reply” based on pilot participant concerns that a fee would be charged for responding to IVR messages.

## Discussion

A theory- and evidence-based approach using qualitative research methods was conducted to design the CHoBI7 mHealth program. The formative research findings have shown that the CHoBI7 mHealth program has high user acceptability and is feasible to deliver to diarrhea patients that present at health facilities for treatment in Bangladesh. Semi-structured interviews with government stakeholders identified their perceptions and preferences for scaling the CHoBI7 mHealth program. mHealth workshops were an interactive approach to draft and refine mobile message content based on stakeholder preferences. Group discussions and semi-structured interviews with diarrhea patients and their family members identified beneficiary perceptions of mHealth and preferences for delivery of the CHoBI7 mHealth program. The pilot of the CHoBI7 mHealth program allowed for an assessment of program feasibility and to identify potential barriers to successful program implementation. Applying the IBM-WASH framework to the development of the CHoBI7 mHealth program allowed us to gain an understanding of the multi-level, multidimensional contextual, psychosocial, and technological factors that were influencing program delivery and the targeted WASH behaviors. This is the first published study, to our knowledge, to conduct formative research to design a WASH mHealth intervention delivered directly to households to increase handwashing with soap and water treatment behavior.

Through government stakeholders interviews we identified contextual and technological factors that will be important to consider for scaling the CHoBI7 mHealth program across Bangladesh. Integration of the CHoBI7 program into existing mHealth programs being delivered by the government, such as mobile messaging promoting child and maternal health and health call centers was recommended by government stakeholders. This could help to reduce the cost of program implementation. Government stakeholders also stated the potential for implementation of the CHoBI7 mHealth program without external donor support, and recommended the CHoBI7 program be included in the upcoming National Operational Plan for the CDC Department. Inclusion of the CHoBI7 program in the National Operational Plan and government funding without external donor support will be crucial to the sustainability of this program if taken to scale across Bangladesh.

Government stakeholders also recommended delivering CHoBI7 mobile messages to high risk populations during the times when they were most susceptible, such as diarrhea outbreaks. This is consistent with the CHoBI7 program approach to initially deliver mobile messages in the health facility to diarrhea patients and their family members when they are at highest risk for diarrhea. This high-risk time is when WASH messages are likely to resonate the most with individuals and facilitate sustained behavior change [36, 40].

There was a positive response for Dr. Chobi as the sender of the CHoBI7 voice and text messages. She was viewed as a credible source of information, with participants reporting they could share the messages that they received from her with others since she was a doctor at the well-known “Cholera Hospital”. Aklima was also well liked, with participants identifying with her as also being a mother who came with her child to the health facility for diarrhea treatment. Through the formative research it emerged that it was important to have the sender of text and voice messages be a known individual and female. This is consistent with a previous study in Ghana that found a positive response with a female voice being used for IVR message delivery [46]. In Bangladesh, Pakistan, and Nepal, the use of the character Meena, a young South Asian girl, for a UNICEF health awareness campaign, delivered by both television and printed communication materials, was immensely successful in increasing handwashing with soap and water treatment [47, 48]. The success of Meena serves as an example of how female characters could be used to facilitate WASH behavior change. Future studies should investigate how the identity of the sender of mobile messages influences behavior for WASH programs.

Both text and voice messages were considered to be important by beneficiaries and government stakeholders for delivery of the CHoBI7 mHealth program. Voice messages were seen as beneficial because they could be understood by those that could not read. While text messages were viewed as being valuable because they could be saved and viewed later and shared messages with others. Text messages were also stated to serve as important reminders to perform the promoted WASH behaviors. This finding lead to the delivery of voice messages followed by a summary text message in the CHoBI7 mHealth program. Most mHealth programs rely on either text or voice message, and rarely employ both [13]. Future studies should investigate preferences around voice and text messages for delivery of WASH programs in other settings.

Gender norms around mobile phone access and message sharing emerged as important contextual and technological factors for delivery of the CHoBI7 mHealth program. Some female household members did not have consistent access to mobile phones, and reported that male household members were not always sharing CHoBI7 mHealth messages. Female participants emphasized the importance of receiving program messages since they were often the ones responsible for taking care of young children, and wanted to keep their child healthy. This is an important challenge to address, particularly if no home visits are conducted to reinforce mHealth messages, and given our setting where female household members have lower rates of phone ownership than male household members. In Bangladesh, it is estimated that 82% of male adults are mobile owners compared to only 55% of adult females [49]. This gender inequity in phone ownership is likely driven by the patriarchal family structure which is common in Bangladesh [50]. The challenge of phone access is consistent with findings from the Aponjon mHealth program [51]. This program in Bangladesh delivers text messages to women during pregnancy on antenatal care. Aponjon subscribers reported that calls were sometimes missed because someone else in the household had the phone. Through the CHoBI7 formative research we were able to identify potential solutions to address the challenge of message sharing and access by delivering messages at a time when most household members were present, sending messages to the phones of both female and male household members (when possible), and through discussing message sharing in the health facility and in mobile message content. The effectiveness of these approaches will be evaluated in our RCT of the CHoBI7 mHealth progarm. These findings will be important given that previous studies have emphasized the importance of usability and accessibility for the effectiveness and sustainability of mHealth interventions [52]. Our results highlight the importance of considering gender dynamics during message development, and the need for intervention approaches that ensure gender equity in access to message content.

An important contextual factor for delivery of text messages for the CHoBI7 program is the literacy rate in the target population. In Bangladesh, the literacy rate for females over 15 years of age is 70%, and 76% for males [53]. Furthermore, in our current work we found that 92% of households in slum areas of Dhaka have at least one person in the home that can read and write (Personal Communication: Christine Marie George). Given this high household literacy rate, text messages presents a feasible approach for intervention delivery in our study setting.

This is the first study, to our knowledge, to use IVR for sending quiz questions to assess knowledge of WASH practices in a low and middle-income country setting. IVR quizzes were well-received by both group discussions and pilot interview participants, with some participants even reporting sharing the content of quiz questions with neighbors. Previous studies have found that two-way text messaging was associated with improved medication adherence practices compared to one-way texting [46, 54]. The current RCT of the CHoBI7 trial will investigate if responding to IVR quiz questions and correct quiz responses are associated with increases in the promoted WASH behaviors and sustained behavior change.

The use of mHealth for promoting WASH behaviors presents a low-cost approach for program delivery that does not involve the cost of frequent in-person visits by health promoters. The VIAMO platform was 0.028 USD per minute for recorded voice messages and 0.041 USD per text message in Bangladesh at the time the pilot study was conducted. Therefore, if text and voice messages are delivered every 2 weeks over a 1 year period, the cost of message delivery would be less than 2 USD per phone. Previous formative research from the Aponjon mHealth program found that the majority of pregnant women and new mothers were willing to pay 0.025 USD per mobile message [51]. Similar research is needed to evaluate willingness to pay for CHoBI7 mHealth messages. Government stakeholders mentioned that the cost could be less for message delivery if mobile messages are sent through the government system. This option should be further explored.

mHealth messages have the potential to serve as important reminders to facilitate WASH behavior change that do not require home visits. However, there are no published studies to date, to our knowledge, that have evaluated the impact of a WASH mHealth programs on WASH behavior change. Previously, mHealth RCTs in low- and middle-income countries have focused mostly on reminders for medication adherence and immunization visits [11–15]. Our current RCT of the CHoBI7 mHealth program will evaluate the impact of this intervention on sustained handwashing with soap and stored drinking water quality.

This study has several strengths. First, the engagement of both government stakeholders and beneficiaries during the formative research that informed intervention development. Through engaging both of these key stakeholders, both scalability and acceptability of the CHoBI7 mHealth program were assessed. Second, the 7-month length of our formative research phase, which allowed for intervention development through an iterative process where changes were made to CHoBI7 mHealth messages based on findings from the pilot study. Third, the use of both group discussions and semi-structured interviews, which provided complementary information. Fourth, the use of mHealth workshops, which were valuable to discuss findings from semi-structured interviews and group discussions, and to draft and refine mobile health message content. Fifth, the use of health theory for intervention development.

One limitation is that this study focused only on households that reported mobile phone ownership, and therefore is not generalizable to households sharing phones with other individuals outside the home. In our current RCT, over 90% of diarrhea patient households screened for eligibility reported household phone ownership (Personal Communication: Christine Marie George). Future studies should evaluate the impact of the CHoBI7 mHealth program on households that report sharing a mobile phone. A second limitation is that the pilot study was conducted only in Dhaka, Bangladesh. Future studies should pilot the CHoBI7 mHealth program across all divisions of Bangladesh. A third limitation is that messages were not sent out using the government’s mobile platform. The VIAMO platform was used given the iterative nature of message development. Future work should use the government’s mobile platform.

## Conclusion

This study presents a theory- and evidence-based approach that can be used for the development of future WASH mHealth programs. The CHoBI7 mHealth program presents a promising scalable approach for which mobile messages can be sent to a large number of households at minimal cost and can serve as valuable cues to action to facilitate WASH behavior change. The ongoing RCT of the CHoBI7 mHealth program is the first to evaluate the impact of voice and text message reminders sent to households promoting handwashing with soap and water treatment behaviors. The evidence generated by this study will be used by the Bangladesh Ministry of Health and Family Welfare to determine the feasibility of scaling the CHoBI7 mHealth program across Bangladesh.

## Additional files

Additional file 1: **Table S1.** Overview of Intervention Activities by Arm. **Table S2.** Mobile Messages Played During the First Group Discussion. **Table S3.** Overview of CHoBI7 Mobile Health Program Development: Themes, Formative Research Findings, and Intervention Components. (DOCX 38 kb)

## Data Availability

The transcripts analyzed during the current study are not publicly available because they contain identifying participant data.

## Abbreviations

CHoBI7: Cholera-Hospital-Based-Intervention-for-7-Days
IBM-WASH: The Integrated Behavioural Model for Water, Sanitation, and Hygiene
icddr,b: International Centre for Diarrhoeal Disease Research, Bangladesh
IVR: Interactive voice response
mHealth: Mobile health
ORS: Oral rehydration solution
RANAS model: Risks, Attitudes, Norms, Abilities, and Self-Regulation model
RCT: Randomized controlled trial
WASH: Water, sanitation, and hygiene

## References

[1] Estimates of global, regional, and national morbidity, mortality, and aetiologies of diarrhoeal diseases (2017). a systematic analysis for the Global Burden of Disease Study 2015. Lancet Infect Dis.

[2] Esrey SA (1996). Water, waste, and well-being: a multicountry study. Am J Epidemiol.

[3] Saha D. Acute diarrhoea in children in rural Gambia: Knowledge, attitude and practice, aetiology, risk factors and consequences among children less than five years of age. Dunedin: The University of Otago; 2013.

[4] George CM, Perin J, Neiswender de Calani KJ, Norman WR, Perry H, Davis TP, Lindquist ED (2014). Risk factors for diarrhea in children under five years of age residing in peri-urban communities in Cochabamba, Bolivia. Am J Trop Med Hyg.

[5] Wolf J, Johnston R, Freeman MC, Ram PK, Slaymaker T, Laurenz E, Pruss-Ustun A. Handwashing with soap after potential faecal contact: Global, regional and country estimates for handwashing with soap after potential faecal contact. Int J Epidemiol. 2018. 10.1093/ije/dyy253. 10.1093/ije/dyy253 PMC6693803 30535198

[6] Halder A, Tronchet C, Akhter S, Bhuiya A, Johnston R, Luby S (2010). Observed hand cleanliness and other measures of handwashing behavior in rural Bangladesh. BMC public health.

[7] Wolf Jennyfer, Hunter Paul R., Freeman Matthew C., Cumming Oliver, Clasen Thomas, Bartram Jamie, Higgins Julian P. T., Johnston Richard, Medlicott Kate, Boisson Sophie, Prüss-Ustün Annette (2018). Impact of drinking water, sanitation and handwashing with soap on childhood diarrhoeal disease: updated meta-analysis and meta-regression. Tropical Medicine & International Health.

[8] Luby SP, Agboatwalla M, Bowen A, Kenah E, Sharker Y, Hoekstra RM (2009). Difficulties in maintaining improved handwashing behavior, Karachi, Pakistan. Am J Trop Med Hyg.

[9] Zwane AP, Kremer M (2007). What works in fighting diarrheal diseases in developing countries? A critical review. World Bank Res Obs.

[10] Qadri F, Ali M, Chowdhury F, Khan AI, Saha A, Khan IA, Begum YA, Bhuiyan TR, Chowdhury MI, Uddin MJ (2015). Feasibility and effectiveness of oral cholera vaccine in an urban endemic setting in Bangladesh: a cluster randomised open-label trial. Lancet.

[11] Zurovac D, Sudoi RK, Akhwale WS, Ndiritu M, Hamer DH, Rowe AK, Snow RW (2011). The effect of mobile phone text-message reminders on Kenyan health workers' adherence to malaria treatment guidelines: a cluster randomised trial. Lancet.

[12] Cole-Lewis H, Kershaw T (2010). Text messaging as a tool for behavior change in disease prevention and management. Epidemiol Rev.

[13] Free C, Phillips G, Galli L, Watson L, Felix L, Edwards P, Patel V, Haines A (2013). The effectiveness of mobile-health technology-based health behaviour change or disease management interventions for health care consumers: a systematic review. PLoS Med.

[14] Higgs ES, Goldberg AB, Labrique AB, Cook SH, Schmid C, Cole CF, Obregon RA (2014). Understanding the role of mHealth and other media interventions for behavior change to enhance child survival and development in low- and middle-income countries: an evidence review. J Health Commun.

[15] Gibson DG, Ochieng B, Kagucia EW, Were J, Hayford K, Moulton LH, Levine OS, Odhiambo F, O'Brien KL, Feikin DR (2017). Mobile phone-delivered reminders and incentives to improve childhood immunisation coverage and timeliness in Kenya (M-SIMU): a cluster randomised controlled trial. Lancet Glob Health.

[16] Key ICT indicators for developed and developing countries and the world (totals and penetration rates) [https://www.itu.int/en/ITU-D/Statistics/Pages/stat/default.aspx]

[17] GSMA (2018). Mobile industry driving growth and enabling digital inclusion.

[18] Curtis V, Schmidt W, Luby S, Florez R, Touré O, Biran A (2011). Hygiene: new hopes, new horizons. Lancet Infect Dis.

[19] Michie S, Prestwich A (2010). Are interventions theory-based? Development of a theory coding scheme. Health Psychol.

[20] Inauen Jennifer, Mosler Hans-Joachim (2013). Developing and testing theory-based and evidence-based interventions to promote switching to arsenic-safe wells in Bangladesh. Journal of Health Psychology.

[21] Contzen N, Meili IH, Mosler HJ (2015). Changing handwashing behaviour in southern Ethiopia: a longitudinal study on infrastructural and commitment interventions. Soc Sci Med.

[22] Taylor N, Conner M, Lawton R (2012). The impact of theory on the effectiveness of worksite physical activity interventions: a meta-analysis and meta-regression. Health Psychol Rev.

[23] Webb TL, Joseph J, Yardley L, Michie S (2010). Using the internet to promote health behavior change: a systematic review and meta-analysis of the impact of theoretical basis, use of behavior change techniques, and mode of delivery on efficacy. J Med Internet Res.

[24] Dreibelbis R, Kroeger A, Hossain K, Venkatesh M, Ram PK (2016). Behavior change without behavior change communication: nudging handwashing among primary school students in Bangladesh. Int J Environ Res Public Health.

[25] Dreibelbis R, Winch PJ, Leontsini E, Hulland KR, Ram PK, Unicomb L, Luby SP (2013). The integrated behavioural model for water, sanitation, and hygiene: a systematic review of behavioural models and a framework for designing and evaluating behaviour change interventions in infrastructure-restricted settings. BMC public health.

[26] Carpenter CJ (2010). A meta-analysis of the effectiveness of health belief model variables in predicting behavior. Health Commun.

[27] Mosler H-J (2012). A systematic approach to behavior change interventions for the water and sanitation sector in developing countries: a conceptual model, a review, and a guideline. Int J Environ Health Res.

[28] Ajzen I (1985). From intentions to actions: A theory of planned behavior: Springer.

[29] Rogers RW (1975). A protection motivation theory of fear appeals and attitude change1. J Psychol.

[30] Contzen N, Mosler HJ (2015). Identifying the psychological determinants of handwashing: Results from two cross-sectional questionnaire studies in Haiti and Ethiopia. Am J Infect Control.

[31] Curtis VA, Danquah LO, Aunger RV (2009). Planned, motivated and habitual hygiene behaviour: an eleven country review. Health Educ Res.

[32] Weil AA, Khan AI, Chowdhury F, LaRocque RC, Faruque A, Ryan ET, Calderwood SB, Qadri F, Harris JB (2009). Clinical outcomes in household contacts of patients with cholera in Bangladesh. Clin Infect Dis.

[33] George CM, Ahmed S, Talukder KA, Azmi IJ, Perin J, Sack RB, Sack DA, Stine OC, Oldja L, Shahnaij M (2015). Shigella Infections in Household Contacts of Pediatric Shigellosis Patients in Rural Bangladesh. Emerg Infect Dis.

[34] Black RE, Merson MH, Rowe B, Taylor PR, Abdul Alim AR, Gross RJ, Sack DA (1981). Enterotoxigenic *Escherichia coli* diarrhoea: acquired immunity and transmission in an endemic area. Bull World Health Organ.

[35] Burrowes V, Perin J, Monira S, Sack DA, Rashid MU, Mahamud T, Rahman Z, Mustafiz M, Bhuyian SI, Begum F (2017). Risk Factors for Household Transmission of Vibrio cholerae in Dhaka, Bangladesh (CHoBI7 Trial). Am J Trop Med Hyg.

[36] Figueroa ME, Kincaid DL, Sobsey N, Clasen T (2007). Social, cultural and behavioral correlates of household water treatment and storage. *Household water treatment and safe storage*.

[37] Amin N, Pickering AJ, Ram PK, Unicomb L, Najnin N, Homaira N, Ashraf S, Abedin J, Islam MS, Luby SP (2014). Microbiological evaluation of the efficacy of soapy water to clean hands: a randomized, non-inferiority field trial. Am J Trop Med Hyg.

[38] George CM, Monira S, Sack DA, Rashid MU, Saif-Ur-Rahman KM, Mahmud T, Rahman Z, Mustafiz M, Bhuyian SI, Winch PJ (2016). Randomized Controlled Trial of Hospital-Based Hygiene and Water Treatment Intervention (CHoBI7) to Reduce Cholera. Emerg Infect Dis.

[39] The Safe Water System: Free Chlorine Testing [http://www.cdc.gov/safewater/chlorine-residual-testing.html]

[40] George CM, Jung DS, Saif-Ur-Rahman KM, Monira S, Sack DA, Rashid MU, Mahmud T, Mustafiz M, Rahman Z, Bhuyian SI (2016). Sustained Uptake of a Hospital-Based Handwashing with Soap and Water Treatment Intervention (Cholera-Hospital-Based Intervention for 7 Days [CHoBI7]): A Randomized Controlled Trial. Am J Trop Med Hyg.

[41] Devine J, Karver J, Coombes Y, Chase C, Hernandez O (2012). Behavioral determinants of handwashing with soap among mothers and caretakers: emergent learning from Senegal and Peru.

[42] Porzig-Drummond R, Stevenson R, Case T, Oaten M (2009). Can the emotion of disgust be harnessed to promote hand hygiene? Experimental and field-based tests. Soc Sci Med.

[43] Aunger R, Schmidt W-P, Ranpura A, Coombes Y, Maina PM, Matiko CN, Curtis V (2010). Three kinds of psychological determinants for hand-washing behaviour in Kenya. Soc Sci Med.

[44] George Christine Marie, Biswas Shwapon, Jung Danielle, Perin Jamie, Parvin Tahmina, Monira Shirajum, Saif-Ur-Rahman K. M., Rashid Mahamud-ur, Bhuyian Sazzadul Islam, Thomas Elizabeth D., Dreibelbis Robert, Begum Farzana, Zohura Fatema, Zhang Xiaotong, Sack David A., Alam Munirul, Sack R. Bradley, Leontsini Elli, Winch Peter J. (2017). Psychosocial Factors Mediating the Effect of the CHoBI7 Intervention on Handwashing With Soap: A Randomized Controlled Trial. Health Education & Behavior.

[45] Etikan I, Musa SA, Alkassim RS (2016). Comparison of convenience sampling and purposive sampling. Am J Theor Appl Stat.

[46] Crowdsourcing citizen feedback on district development in Ghana using interactive voice response surveys. Making All Voices Count Practice Paper. Brighton: Institute of Development Studies [http://opendocs.ids.ac.uk/opendocs/handle/123456789/12716]

[47] Chesterton P. Evaluation of the Meena communication initiative. Kathmandu: UNICEF; 2004. https://www.unicef.org/evaldatabase/files/ROSA_2004_800_Meena_Comm_Initiative.pdf

[48] Anis Ferdousi, White Julie (2017). The Meena Communicative Initiative in Bangladesh. Inclusive Education.

[49] Rowntree O. The Mobile Gender Gap Report 2018: GSMA, Retrieved from https://www.gsma.com/mobilefordevelopment/programmes/connected-women/the-mobile-gender-gap-report-2018; 2018.

[50] Kabeer N (2011). Between affiliation and autonomy: navigating pathways of women's empowerment and gender justice in rural Bangladesh. Dev Chang.

[51] Rajan R, Raihan A, Alam M, Agarwal S, Ahsan A, Bashir R, Lefevre A, Kennedy C, Labrique A (2013). MAMA ‘Aponjon’formative research report.

[52] Aranda-Jan CB, Mohutsiwa-Dibe N, Loukanova S (2014). Systematic review on what works, what does not work and why of implementation of mobile health (mHealth) projects in Africa. BMC Public Health.

[53] Bangladesh. UNESCO.

[54] Wald DS, Butt S, Bestwick JP (2015). One-way versus two-way text messaging on improving medication adherence: meta-analysis of randomized trials. Am J Med.

