# Correction to: Formative research for the design of a scalable water, sanitation, and hygiene mobile health program: CHoBI7 mobile health program

**DOI:** 10.1186/s12889-019-7477-7

**Published:** 2019-08-19

**Authors:** Christine Marie George, Fatema Zohura, Alana Teman, Elizabeth Thomas, Tasdik Hasan, Sohel Rana, Tahmina Parvin, David A. Sack, Sazzadul Islam Bhuyian, Alain Labrique, Jahed Masud, Peter Winch, Elli Leontsini, Kelsey Zeller, Farzana Begum, Abul Hasem Khan, Sanya Tahmina, Farazana Munum, Shirajum Monira, Munirul Alam

**Affiliations:** 1 Department of International Health, Program in Global Disease Epidemiology and Control, Johns Hopkins Bloomberg School of Public Health, 615 N. Wolfe Street, Room E5535, Baltimore, MD 21205-2103 USA; 2 International Centre for Diarrhoeal Disease Research, Bangladesh (icddr,b), Dhaka, Bangladesh; 3 Ministry of Health and Family Welfare, Dhaka, Bangladesh

**Correction to: BMC Public Health**

**http://dx.doi.org/10.1186/s12889-019-7144-z**

It was highlighted that the original article [1] contained an error in the title. Additionally, Table 2 contained a typesetting mistake. This Correction article shows the incorrect and correct article title and Table 2. The original article has been updated.

The Publisher apologizes to the authors and readers for the inconvenience caused by the typesetting mistake.

**Incorrect title:**

Formative research for the design of a scalable mobile health program water, sanitation, and hygiene: CHoBI7 mobile health program.

**Correct title:**

Formative research for the design of a scalable water, sanitation, and hygiene mobile health program: CHoBI7 mobile health program.

Incorrect Table 2 (affected section underlined):

Correct Table 2 (affected section underlined):

**Table 2:** The IBM-WASH Framework Applied to the Development of the CHoBI7 Mobile Health Intervention Based on Qualitative Findings

BM-WASH Dimension	Contextual Factors	Psychosocial Factors	Technological Factors
Structural/ Societal	• Existing government mobile health programs send out health-related messages on government health days, these include voice and text mobile messages • Potential inclusion of CHoBI7 intervention in the National Operational Plan in Bangladesh • Potential integration of CHoBI7 in existing mobile health programs in Banglades	• Government commitment to mobile health as a method to deliver public health information	• Existing government mobile platform has the potential to be used for CHoBI7 intervention delivery at reduced cost • Health Education Bureau in the Ministry of Health and Family Welfare currently develops mobile health messages, and can be potentially engaged for CHoBI7 intervention development
Community	• High household mobile phone access and ownership in Bangladesh	• Sharing of mHealth messages with neighbors	• High mobile network coverage in Dhaka, Bangladesh
			• Most feature phones available in Bangladesh allow for viewing of Bangla script
Interpersonal	• Females in the households are often the ones responsible for caring for young children • Male household members may not want female household members to receive text and voice messages from an unknown sender	• Female caregivers requesting access to CHoBI7 mHealth messages to allow them to better care for their children	• Text messages allow for sharing with others at a later time • Male household members do not always share mobile messages or their mobile phones with other household members • Timing for mobile message delivery when all household members are present • Adult male household members typically have primary mobile phone ownership in household • Lower female access to mobile phones
Individual	• Literacy rate of household members • Limited mobile message sharing by those working outside of the home	• Self-efficacy to open text messages, and respond to interactive voice response messages	• Concerns about being charged a fee for viewing or listening to mobile messages
Habitual	• Frequency of exposure to mobile messages	• Outcome expectancy that following recommendations contained in mobile messages will reduce disease	• Voice and text messages as reminders to perform the promoted water, sanitation, and hygiene behaviors

**Table 2:** The IBM-WASH Framework Applied to the Development of the CHoBI7 Mobile Health Intervention Based on Qualitative Findings

IBM-WASH Dimension	Contextual Factors	Psychosocial Factors	Technological Factors
Structural/ Societal	• Existing government mobile health programs send out health-related messages on government health days, these include voice and text mobile messages • Potential inclusion of CHoBI7 intervention in the National Operational Plan in Bangladesh • Potential integration of CHoBI7 in existing mobile health programs in Bangladesh	• Government commitment to mobile health as a method to deliver public health information	• Existing government mobile platform has the potential to be used for CHoBI7 intervention delivery at reduced cost • Health Education Bureau in the Ministry of Health and Family Welfare currently develops mobile health messages, and can be potentially engaged for CHoBI7 intervention development
Community	• High household mobile phone access and ownership in Bangladesh	• Sharing of mHealth messages with neighbors	• High mobile network coverage in Dhaka, Bangladesh
			• Most feature phones available in Bangladesh allow for viewing of Bangla script
Interpersonal	• Females in the households are often the ones responsible for caring for young children • Male household members may not want female household members to receive text and voice messages from an unknown sender	• Female caregivers requesting access to CHoBI7 mHealth messages to allow them to better care for their children	• Text messages allow for sharing with others at a later time • Male household members do not always share mobile messages or their mobile phones with other household members • Timing for mobile message delivery when all household members are present • Adult male household members typically have primary mobile phone ownership in household • Lower female access to mobile phones
Individual	• Literacy rate of household members • Limited mobile message sharing by those working outside of the home	• Self-efficacy to open text messages, and respond to interactive voice response messages	• Concerns about being charged a fee for viewing or listening to mobile messages
Habitual	• Frequency of exposure to mobile messages	• Outcome expectancy that following recommendations contained in mobile messages will reduce disease	• Voice and text messages as reminders to perform the promoted water, sanitation, and hygiene behaviors
