# Supplementary material for: Formative research for the design of a scalable mobile health program water, sanitation, and hygiene: CHoBI7 mobile health program
Source: BMC Public Health. 2019 Jul 31;19:1028. doi: 10.1186/s12889-019-7144-z (PMC6670164; doi:10.1186/s12889-019-7144-z)
Supplement: Supplementary file 1 — Table S1. Overview of Intervention Activities by Arm. Table S2. Mobile Messages Played During the First Group Discussion. Table S3. Overview of CHoBI7 Mobile Health Program Development: Themes, Formative Research Findings, and Intervention Components. (DOCX 38 kb) [file 12889_2019_7144_MOESM1_ESM.docx]

| **Supplementary Table 1**. Overview of CHoBI7 mHealth Program Pilot Activities by Arm   \|  \| **Standard Recommendation Arm** \| **mHealth with no Home Visits**  **Arm** \| **mHealth with Home Visits**  **Arm** \| \| --- \| --- \| --- \| --- \| \| **Intervention Visits and**  **Phone Messages** \| 1 Health Facility Visit \| 1 Health Facility Visit &  CHoBI7 Mobile Health Program \| 1 Health Facility Visit  &  2 Home Visits  &  CHoBI7 Mobile Health Program \| \| **Intervention Activities** \| - Promoter delivers the standard recommendation on the use of oral rehydration solution (ORS) for dehydration in the health facility \| - Promoter delivers the standard recommendation on the use of ORS for dehydration in the health facility. - Promoter delivers the CHoBI7 flipbook module and diarrhea prevention package in the health facility to the diarrhea patient and their household members. - Voice and text messages are sent to households by the CHoBI7 mHealth program bi-weekly for 1 year. \| - Promoter delivers the standard recommendation on the use of ORS for dehydration in the health facility. - Promoter delivers the CHoBI7 flipbook module and diarrhea prevention package in the health facility to the diarrhea patient and their household members. - Promoter visits the diarrhea patient household twice to deliver the CHoBI7 flipbook module (home visits) during the first week after the diarrhea patient is admitted to the health facility. - Voice and text messages are sent to households by the CHoBI7 mHealth program bi-weekly for 1 year. \|      \| **Supplementary Table 2.** CHoBI7 mHealth Program Mobile Messages Played During the First Group Discussion \| \| \| \| --- \| --- \| --- \| \|  \| **Message Type and Topic** \| **Content** \| \| **Message 1** \| Voice: Introduction of characters, 7-day high-risk period for diarrhea transmission, intervention hardware setup \| Assalamulalaikum, my name is Dr. Chobi apa from the Mohakhali Cholera Hospital. I’m going to be speaking with you regularly over the next few months about handwashing with soap and water treatment, to help remind you how to keep your family safe from more severe diarrhea!  All of my calls and messages will be free.  Right now and for the next 7 days, your family is at a very high risk for getting severe diarrhea again – I want to share information with you to help prevent this!  You may have just gotten home from the hospital, where my CHoBI7 staff gave you a handwashing station and safe water storage bucket. Have you set up the water bucket on a stool, table or other high place? Add the chlorine tablet and cover it with the lid!  Also make sure the handwashing station is in a convenient place for all your family to use! It should be sitting on the provided stool, and the soapy water bottle should be next to it.  I’ll talk to you again tomorrow, take care! \| \| **Message 2** \| Voice: Introduction of characters, 4 key times for hand washing with soap \| Assalamulalaikum, this is Dr. Chobi apa from Mohakhali Cholera Hospital calling again. I hope that your family is feeling well. I am with Aklima. We have been discussing how important washing your hands with soap is.  An upsetting thing is that severe diarrhea comes from germs in feces, and spreads through hands and water.  To protect your family from these germs, make sure your whole family washes their hands with soap at 4 key times:  After defecation, after cleaning child feces and a child’s anus, before preparing food and before eating or feeding children. Don’t forget to use soapy water each time, and ask your family members to do the same!  Aklima, Can you tell me about the importance of washing your hands with soapy water?  Aklima: Yes! Washing with soapy water keeps my hands free from germs so that my family can stay healthy!  I also know how to wash my hands properly with soap. First, I must wet my hands and rub them together with soapy water. Then I rub my hands, palm and surface, between my fingers and under the nails! I keep this up for 20 seconds. Now I’m done, and just rinse my hands with water and dry them with a clean dry towel.  Dr. Chobi: Thank you, Aklima! That’s absolutely right! Thank you for reviewing that with me. You and Aklima are starting to become experts on these things. I’ll send you some SMS over the next few days to help you remember what we’ve already spoken about.  Remember, the health of your family is in your hands! \| \| **Message 3** \| Text: Water treatment \| Your water may look clean but how do you know if it is free of germs? When you boil your water then you know. \| \| **Message 4** \| Text: Disgust \| If you don’t wash your hands with soap after using the toilet, you’re eating poop, when you’re eating rice. There are lots of germs in the poop, and you will have diarrhea again. \|   **Supplementary Table 3.** Overview of CHoBI7 Mobile Health Program Development: Themes, Formative Research Findings, and Intervention Components | | |
| --- | --- | --- | --- | --- | --- | --- | --- | --- | --- | --- | --- | --- | --- | --- | --- | --- | --- | --- | --- | --- | --- | --- | --- | --- | --- | --- | --- | --- | --- | --- | --- | --- |
| **Theme** | **Formative Research Finding** | **Intervention Component** |
| **Mobile Message Delivery Preference (Voice or Text)** | **Government Stakeholders:** Positive response for both voice and text messages. Voice messages were stated to be more easily understood by those who could not read. Text messages were stated to have been used in previous government programs. | A combination of voice and text messages are sent, and a summary text message was sent out after each voice message. |
|  | **Group Discussions:** Both voice and text messages were recommended. Text messages that summarized the content of voice messages were recommended. |  |
|  | **Pilot Interviews:** Voice messages were preferred. Some recommended a combination of voice and text messages, and sending text and voice messages together. |  |
| **Target Population for Message Delivery** | **Government Stakeholders:** Recommended messages should be sent to high-risk populations, such as populations impacted by diarrhea outbreaks. | Messages are sent to households of diarrhea patients. |
| **Script or Phonetic Bangla for Text Messages** | **Government Stakeholders:** Phonetic Bangla was recommended. | Bangla script is used for all text messages. |
|  | **Group Discussions:** Bangla Script was recommended |  |
|  | **Pilot Exit Interviews:** Bangla script was recommended. |  |
| **CHoBI7 Message Sender:**  **Dr. Chobi and Aklima** | **Government Stakeholders**: The following individuals were recommended: Prime Minster, Health Minister, Parliament Member, health provider, socialite, elite person, high level person from icddr,b, or celebrities such as a cricket player or singer. A female voice was recommended. | A female health care provider from icddr,b Cholera Hospital,  Dr. Chobi, was selected to be the sender of voice and text messages. Aklima was also created to be a mother of a child that came to this hospital with diarrhea. |
|  | **Group Discussions:** Participants liked receiving messages from icddr,b Cholera Hospital. Dr. Chobi was well-received. The female persona of Dr. Chobi in her 30s was preferred. Aklima was also well-received and participants related to her. |  |
|  | **Pilot Interviews:** Dr. Chobi and Aklima were both well-received. |  |
| **Caller ID for Message Sender** | **Group Discussions:** Recommended Dr. Chobi's name be shown on the caller ID. | Health promoters help diarrhea patients and their family members to save the number for Dr. Chobi in their phones when they are at the health facility. All text messages show Dr. Chobi as the sender, and individuals are shown how to open text messages by a promoter in the health facility. |
|  | **Pilot Interviews:** It was stated that husbands may be suspicious of wives if an unknown person calls or sends them a text message. One participant stated that some people may not receive our voice calls because they may think they are advertisements from companies. |  |
| **Message Content** | **Government Stakeholders:**  A few key messages that were very specific and based on scientific evidence were recommended. | Voice messages were shorten to approximately two minutes. |
|  | **Group Discussions:** Message content was clear. Voice messages were too long. |  |
|  | **Pilot Interviews:** Message content was stated to be clear. One participant stated voice messages should be longer and another participant stated text messages should be longer. |  |
| **Target Person for Message Delivery/ Access to CHoBI7 mHealth Messages** | **Group Discussion:** Participants recommended mobile messages be sent to the husband and wife. Female participants emphasized the importance of messages coming to them since they were often the primary caregiver in the home. One participant expressed concern about messages being sent to his wife's phone. | During health facility delivery of the CHoBI7 program, health promoters ask diarrhea patients and their family members whom in the household should receive CHoBI7 mobile messages. The importance of female household members receiving CHoBI7 mHealth messages for the health of their children is discussed, and it is stated that a female doctor from icddr,b Cholera Hospital would be contacting them. |
|  | **Pilot Interviews:** Some female participants reported not having their own mobile phone, and some stated that male household members did not always share mobile messages from the CHoBI7 program with them. It was recommended that CHoBI7 messages be sent to the female household members who were caring for children. One participant stated that husbands may be suspicious of wives if they receive phone messages from an unknown sender. |  |
| **Sharing Mobile Messages with Others** | **Group Discussion:** Participants stated sharing mobile messages from Dr. Chobi with spouses, neighbors, and family members. | All text and voice messages state "Please share this message." |
|  | **Pilot Interviews:** Participants stated sharing messages with spouses, relatives, and friends. |  |
| **Timing of Mobile Message Delivery** | **Group Discussions:** Late afternoon, evening, or night was preferred. | Text and voice messages are sent out at 5 PM in the afternoon. |
|  | **Pilot Interviews:** Evening time was preferred by the most participants. |  |
| **Timing and frequency for CHoBI7 mHealth message delivery** | **Government Stakeholders:** One respondent recommended sending phone messages out every two weeks for three months. | Voice and text messages are sent at least once every two weeks for 1 year. |
|  | **Group Discussions:** Weekly sessions were recommended with Dr. Chobi. |  |
|  | **Pilot Interviews:** Most pilot participants wanted to receive phone messages weekly, and all stated at least once per month. It was recommended that messages be sent out for 6 months to 1 year. |  |
| **Replying to Interactive Voice Response (IVR) Messages** | **Group Discussions:** Participants described previous experiences of unknowingly being charged for text messages from health information lines and herbal companies. | All IVR messages state "There is no charge for your reply." A tutorial was also given by a health promoter in the health facility on how to respond to an IVR (quiz) message and how to open text messages. |
|  | **Pilot Interviews**: One participant stated that sometimes a fee is charged for listening to voice messages so it is important to state in CHoBI7 messages that no fee will be charged for listening. |  |
